# Global insights into traumatic brain injury. The low- and middle-income countries’ perspective

**DOI:** 10.62675/2965-2774.20250255

**Published:** 2025-06-30

**Authors:** Cássia Righy, Carla Bittencourt Rynkowski, Ricardo Turon

**Affiliations:** 1 Fundação Oswaldo Cruz Intensive Care Medicine Laboratory Rio de Janeiro RJ Brazil Intensive Care Medicine Laboratory, Fundação Oswaldo Cruz - Rio de Janeiro (RJ), Brazil.; 2 Instituto Estadual do Cérebro "Paulo Niemeyer" Rio de Janeiro RJ Brazil Instituto Estadual do Cérebro "Paulo Niemeyer" - Rio de Janeiro (RJ), Brazil.; 3 Universidade Federal de Ciências da Saúde de Porto Alegre Faculdade de Medicina Department of Emergency and Trauma Porto Alegre RS Brazil Department of Emergency and Trauma, Faculdade de Medicina, Universidade Federal de Ciências da Saúde de Porto Alegre - Porto Alegre (RS), Brazil.; 4 Hospital Cristo Redentor Intensive Care Unit Porto Alegre RS Brazil Intensive Care Unit, Hospital Cristo Redentor - Porto Alegre (RS), Brazil.; 5 Hospital Niteroi D’Or Niteroi RJ Brazil Hospital Niteroi D’Or - Niteroi (RJ), Brazil.

## INTRODUCTION

Traumatic brain injury (TBI) is a significant public health problem, affecting approximately 55 to 69 million people annually. Despite being largely preventable, TBI impacts particularly in low- and middle-income countries (LMICs), with more than 70% of trauma-related deaths.^(1-3)^

Although often grouped, there are significant differences between low and middle-income countries. According to the World Bank, low-income countries (LIC) have a Gross National Income (GNI) per capita of $1,145 or less, while middle-income countries have a GNI per capita between $1,136 and $13,845 (https://datahelpdesk.worldbank.org/). Middle-income countries (MICs) account for one-third of global gross domestic product (GDP) and are key drivers of global economic growth. These economic distinctions create substantial heterogeneity among the nations collectively referred to as LMICs. This paper focuses on MICs, home to 75% of the world's population and 62% of its poor.

### Understanding the problem

Road traffic accidents are the leading cause of TBI in MICs, particularly affecting young males with moderate to severe TBI, such as extradural hematoma.^(4)^ A nationwide study of TBI patients in Brazil revealed an incidence rate of 65.54 per 100,000 inhabitants between 2008 and 2019, with young males being the most affected (age 20 - 39 years and a 3.6 male-to-female ratio).^(5)^ Similarly, a nationwide registry of TBI patients in Ecuador indicated an overall incidence rate of 154.2 per 100,000 between 2004 and 2016.^(6)^ Notably, there has been a decreasing trend in Ecuador since 2011, attributed to the enforcement of alcohol restriction laws.^(6)^

**Figure 1:**
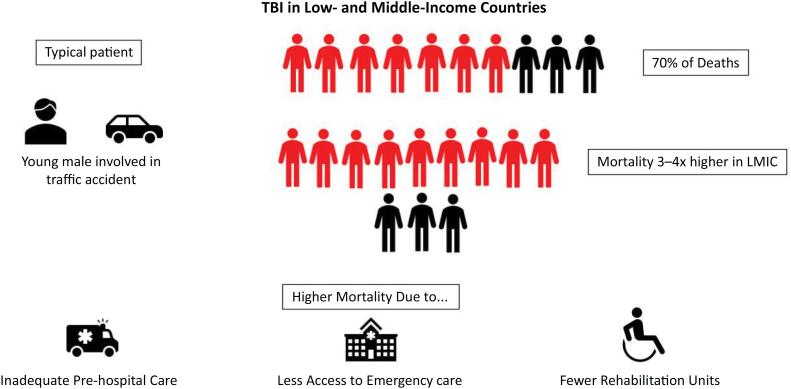
The low- and middle-income countries actual condition.

Middle-income countries generally have higher recorded incidence of TBI compared to LICs, partially due to better hospital registries.^(7)^ Factors such as difficult access to neurotrauma care, inadequate healthcare infrastructure, lack of financial resources, and a shortage of trained healthcare professionals contribute to the poorer outcomes of TBI.^(4)^ Studies show that while 77.3% of the population in high-income countries (HICs) lives within 2 hours of neurotrauma care, only 44.9% of the population in MICs and 25% in LICs have similar access^(8)^ (Figure 1). Additionally, significant challenges exist in accessing healthcare and rehabilitation services. There are also notable disparities within these countries, with some areas suffering from an even greater scarcity of specialized physicians and healthcare infrastructure. This lack of resources leads to higher mortality rates and long-term disabilities^(9)^ (Figure 1).

TBI - traumatic brain injury; LMIC - low- and middle-income countries.

Furthermore, despite the high burden of TBI, research output in MICs is limited due to barriers such as insufficient funding and lack of access to scientific publications. This limited research output hampers the development of context-specific treatment protocols and the overall improvement of TBI care in these regions.

## STEPS FORWARD

To tackle the TBI burden in LMICs, it is essential to understand the local characteristics and scale of the problem. Local health leaders need official epidemiological data to recognize TBI as a significant defiance and effectively guide policy strategies. Resource allocation should focus not only on preventive measures but also on hospital care and rehabilitation tailored to local needs.^(10)^

For instance, in a small, impoverished city in Northeast Brazil, motorcycles are the primary means of transportation, with multiple passengers often riding without helmets. This local context demands more than a standard helmet-wearing campaign; it requires tailored strategies to mitigate the TBI burden effectively.

Besides TBI's principal mechanism and severity, it is fundamental to know the patient's outcomes, which are the leading causes of TBI mortality. Also, the survivors need a rehabilitation plan adapted to the local reality.

## ROADMAP FOR RESEARCH

Research on TBI in LMICs demands an understanding of the unique contexts within these countries. It is essential to craft research agendas and strategic plans that align with governmental priorities while addressing the needs of patients and their families.^(11)^ Research agendas should emphasize critical areas such as healthcare infrastructure and resource allocation for TBI. Insights into TBI's burden, causes, and outcomes in LMICs are vital for developing effective interventions.

Evaluating existing healthcare infrastructure, including pre-hospital care, emergency services, surgical capacity, and rehabilitation facilities, can identify gaps and areas needing improvement.^(12,13)^ Leveraging data from national and regional TBI registries can provide insights into specific gaps. Aligning these data-driven insights with resource allocation strategies will help LMICs prioritize interventions with the most significant potential to improve patient outcomes.^(13)^

Guidelines developed in HIC are not easily reproducible in resource-limited settings. Therefore, international TBI guidelines must be adapted to fit local contexts. The BEST TRIP study shows that treating intracranial hypertension without intracranial pressure (ICP) monitoring is a feasible alternative.^(14)^ Another example is the CREVICE (Consensus-Based Management Protocol for the Treatment of Severe Traumatic Brain Injury),^(15)^ constructed for use when ICP monitoring is unavailable, which is often the case in most MICs.

Empowering local healthcare professionals and researchers is central to improving TBI outcomes in LMICs. Establishing and supporting training programs in TBI management and research methodologies can significantly impact patient care. Fostering partnerships between local institutions and international organizations can facilitate sharing valuable insights and collaboration toward common goals. Investing in research infrastructure, such as laboratories and data management systems, will lay a strong foundation for these efforts. Additionally, data collection and sharing among countries must be prioritized to build research capacity in LMICs.

Above all, we must ensure that TBI research is well-funded and sustainable, seeking out diverse funding sources, including government grants, international aid, private sector investments, and philanthropic contributions. By conducting cost-effectiveness analyses, we can highlight the real value of TBI interventions and attract the necessary financial support. Developing long-term research agendas and strategic plans will help maintain momentum and ensure that our efforts lead to lasting improvements in patient outcomes in MICs.

## CONCLUSION

Traumatic brain injury presents a major significant public health challenge in middle-income countries, where limited healthcare infrastructure, economic challenges, and socio-cultural factors contribute to poorer outcomes. To improve traumatic brain injury care, it is essential to enhance requires enhancing data collection, tailor clinical guidelines to local needs, and strengthen healthcare systems through targeted investments and capacity building. A comprehensive approach includes culturally sensitive prevention, acute care, and rehabilitation. A coordinated global response that prioritizes the needs of middle-income countries is essential to reduce the burden of traumatic brain injury and improve patient outcomes.
